# Challenges in employing overall survival as a primary outcome in ovarian cancer studies: insights from PARP inhibitor trials

**DOI:** 10.3389/fonc.2026.1803208

**Published:** 2026-03-11

**Authors:** Yoav Siegler, Emad Matanes

**Affiliations:** Department of Obstetrics and Gynecology, Rambam Health Care Campus, Haifa, Israel

**Keywords:** biologic treatment effectiveness, clinical trials, ovarian cancer, overall survival, PARP inhibitors

On April 2020, the U.S. Food and Drug Administration (FDA) approved niraparib (ZEJULA, GlaxoSmithKline) as maintenance therapy after first line of chemotherapy, for adults with advanced epithelial ovarian, fallopian tube, or primary peritoneal cancer, following the PRIMA trial, which demonstrated a significant improvement in progression-free survival (PFS) (1, 2). However, four years later, the final overall survival (OS) data revealed no difference between the treatment and control groups, even in the presence of homologous recombination deficiency (3). In light of this discrepancy, one might question whether the treatment is truly effective, whether one endpoint is superior to another, or whether greater emphasis should instead be placed on critically examining the clinical outcomes commonly used in oncologic studies and their limitations.

For more than two decades, the standard treatment for advanced-stage ovarian cancer consisted of cytoreductive surgery combined with systemic platinum-based chemotherapy, most commonly carboplatin plus paclitaxel. Despite this approach, recurrence rates remain high, approaching 80%—and the 5-year survival rate is poor, at approximately 30% (4, 5). These limitations highlighted the urgent need to develop effective maintenance therapies that improve long-term outcomes while preserving patients’ well-being and overall quality of life. Dramatic advancements in the treatment of advanced ovarian cancer over the past decade, largely driven by the clinical introduction of PARP inhibitors.

Initially approved as maintenance therapy for recurrent platinum-sensitive disease, PARP inhibitors later became standard maintenance treatment in the first-line setting for patients or tumors with BRCA mutations (germline or somatic) or tumors with a homologous recombination deficiency (HRD) profile based on its positive results in terms of progression free survival (PFS) in three pivotal Phase 3 trials: PRIMA, SOLO-1, and PAOLA-1 (1, 6–11).

PRIMA/ENGOT-OV26/GOG-3012 trial, is the most recent to publish final OS data in September 2024. With a median follow up of 73.9 months, this trial has failed to demonstrate improvement in overall survival with a hazard ratio (HR) of 1.01 [95% confidence interval (CI) 0.84-1.23; P = 0.8834] for niraparib versus placebo in the general population and hazard ratio of 0.93 (95% CI 0.69-1.26) for patients with HRD tumor (3, 10).

The SOLO1/GOG 3004 trial has demonstrated a similar pattern. Initial results, published in 2018, showed a significant improvement in PFS for patients with newly diagnosed advanced ovarian cancer and a BRCA1/2 mutation who received maintenance olaparib compared to placebo. With a median follow-up of 41 months, the trial reported an HR for disease progression or death of 0.30 (95% CI, 0.23–0.41; p < 0.001) (9). However, the final OS results, published in September 2022 after a 7-year follow-up, revealed an HR of 0.55 (95% CI, 0.40–0.76; p = 0.004), which, despite being clinically meaningful, did not meet the trial’s predefined threshold for statistical significance (12).

Similarly, the PAOLA-1/ENGOT-ov25 trial showed a significant improvement in PFS, with an HR of 0.59 (95% CI, 0.49–0.72; p < 0.001). However, no OS benefit was observed in the general population after a median follow-up of 61 months (HR 0.92, 95% CI 0.76–1.12; p = 0.4118). Notably, in the HRD-positive subgroup, OS was prolonged with the combination of olaparib and bevacizumab (HR 0.62, 95% CI 0.45–0.85), with a 5-year OS rate of 65.5% compared to 48.4% (11).

These findings prompt an important question: how can trials demonstrating such striking early improvements in PFS fail to show comparable benefits in what is arguably a more clinically meaningful endpoint—overall survival?

There are several suggested explanations to answer this question:

1- Ovarian cancer is characterized by a prolonged disease course, often involving multiple lines of therapy, varying treatment combinations, and crossover between treatment arms—factors that make it difficult to isolate the direct effect of the studied intervention on OS (13, 14).

Specifically, in PARP inhibitor trials—where the drugs are typically used as maintenance therapy following a response to chemotherapy—it is challenging to attribute improvements in OS solely to the investigational treatment. This is because many patients receive PARP inhibitors later in the disease course, either off-trial or as part of subsequent lines of therapy. For example, 44% of patients in the placebo arm in the SOLO1 trial were eventually treated with PARPi during subsequent lines (12). Similar results of subsequent PARPi treatment were reported both in the PRIMA trial and the PAOLA-1 trial with 37% and more 45% of patient in the placebo arm eventually receiving PARPi as subsequent treatment, respectively (3, 11). This treatment crossover, blurs differences between study arms and undermines the validity of OS as a definitive outcome.

2- Significant differences exist among the three Phase 3 trials in terms of patient characteristics. SOLO-1 included only BRCA-mutated patients who achieved complete or partial responses to platinum-based chemotherapy. PAOLA-1 enrolled a broader population regardless of BRCA status, with stratification according to HRD status. PRIMA included the most heterogeneous population, encompassing diverse BRCA/HRD profiles and a higher proportion of high-risk patients (e.g., stage IV disease, partial responders, prior neoadjuvant therapy, or residual disease).

Such heterogeneity can substantially influence treatment response, complicate interpretation of outcomes, and limit cross-trial comparability. Overall survival is particularly sensitive to this variability, as it is affected not only by the study drug but also by baseline prognosis, subsequent therapies, and differences in disease biology across patient subgroups.

3- All these trials were primarily designed and statistically powered to detect differences in PFS, not OS. Demonstrating an OS benefit would require larger sample sizes and significantly longer follow-up—logistical and financial challenges in modern clinical research.

4- Demonstrating a significant difference in OS often requires very long follow-up periods, which delays the evaluation and potential integration of new therapies into clinical practice. As a result, surrogate endpoints such as “Progression free survival” (PFS) and “Time to subsequent therapy” (TFST) have become more commonly used in these studies. These endpoints are more sensitive to the intervention, measurable earlier, and less influenced by post-progression therapies.

Although it is challenging to demonstrate a statistically significant improvement in OS in upfront advanced ovarian cancer treatment studies, a comparison of survival data from recent trials involving PARP inhibitors with those conducted prior to the introduction of these agents indicates a substantial improvement in the life expectancy of women treated with PARP inhibitors. Despite the inherent limitations of cross-trial comparisons, a notable improvement in OS has been observed in patients with high-risk disease enrolled in the PAOLA-1 (42 months) and PRIMA (48 months) trials, where maintenance therapy with PARP inhibitors was administered. In contrast, the ICON7 study for instance (15), which utilized bevacizumab alone for maintenance in high-risk population, reported a shorter OS of 30 months.

Elbaz et al. examined FDA oncology drug approvals and reported that between 2006 and 2023, 31% demonstrated a significant overall survival benefit—22% at the time of initial approval and an additional 8% in subsequent analyses (16). Alternative research endpoints have been proposed and increasingly adopted in recent studies. Endpoints such PFS2 (time to second progression) better capture the long-term impact of initial therapy on subsequent treatment lines and, in certain settings, correlate more closely with overall survival than PFS. Overall response rate (ORR), commonly used in small, single-arm trials, provides direct evidence of antitumor activity through tumor shrinkage, although it often does not translate into survival benefit or improved quality of life. Patient-reported outcomes focusing on quality of life have therefore become central to contemporary research, as they reflect the patient’s perspective on symptoms and functional status. Together, these endpoints help ensure that clinical benefits translate into meaningful patient experiences and that potential survival gains are not offset by treatment-related toxicity (17, 18). Recent studies have also suggested intrinsic tumor cell response and T cell immunoreactivity as beneficial predictive biomarkers for treatment success (19–21).

PARP inhibitors raise important economic and safety considerations that support a measured and individualized approach to their clinical use. Cost-effectiveness analyses consistently show that, at current prices, these agents frequently do not meet accepted economic thresholds, particularly when used as maintenance therapy in the recurrent disease setting (22–25). In parallel, PARP inhibitors are associated with a characteristic class-effect toxicity profile, dominated by hematologic adverse events such as anemia, leukopenia, neutropenia, and thrombocytopenia, along with gastrointestinal symptoms including nausea, vomiting, constipation, and diarrhea (26–29). These adverse effects commonly necessitate dose reductions or temporary treatment interruptions, with more than half of treated patients requiring some form of dose modification, potentially influencing overall treatment value and patient experience (26–29).

In non-BRCA populations, the clinical role of PARP inhibitors is more nuanced and reflects a balance between modest efficacy, toxicity, and cost. Evidence indicates reduced but measurable benefit in ovarian cancers lacking BRCA1/2 mutations or homologous recombination deficiency (HRD). A large meta-analysis of randomized trials demonstrated a statistically significant, though attenuated, progression-free survival (PFS) benefit in non-BRCA and HRD-negative subgroups compared with BRCA-mutated or HRD-positive disease (28). However, individual patient data analyses in newly diagnosed advanced disease suggest that truly HR-proficient tumors derive little clinically meaningful benefit (30). Recent meta-analyses confirm a gradient of benefit favoring BRCA-mutated and HRD-positive tumors, alongside increased rates of high-grade hematologic toxicity (29). Consequently, health-economic evaluations and expert reviews generally support prioritizing PARP inhibitors for biomarker-defined populations, while recommending cautious, selective use in biomarker-negative disease (22–24).

In summary, although overall survival (OS) is regarded as a “hard” and objective endpoint, in the context of ovarian cancer—and especially in trials involving PARP inhibitors—it may not fully or promptly capture the clinical benefit of treatment.

We do not believe these limitations reflect a lack of drug efficacy; rather, they highlight the challenges of using overall survival as the sole valid endpoint. This underscores the importance of selecting alternative, contextually appropriate endpoints that are better aligned with the specific clinical setting and therapeutic goals.
